# The Roots of Bioinformatics

**DOI:** 10.1371/journal.pcbi.1000809

**Published:** 2010-06-24

**Authors:** David B. Searls

**Affiliations:** Independent Consultant, Philadelphia, Pennsylvania, United States of America

## Introduction

Every new scientific discipline or methodology reaches a point in its maturation where it is fruitful for it to turn its gaze inward, as well as backward. Such introspection helps to clarify the essential structure of a field of study, facilitating communication, pedagogy, standardization, and the like, while retrospection aids this process by accounting for its beginnings and underpinnings.

In this spirit, *PLoS Computational Biology* is launching a new series of themed articles tracing the roots of bioinformatics. Essays from prominent workers in the field will relate how selected scientific, technological, economic, and even cultural threads came to influence the development of the field we know today. These are not intended to be review articles, nor personal reminiscences, but rather narratives from individual perspectives about the origins and foundations of bioinformatics, and are expected to provide both historical and technical insights. Ideally, these articles will offer an archival record of the field's development, as well as a human face on an important segment of science, for the benefit of current and future workers.

Upcoming articles, already commissioned, will cover the roots of bioinformatics in structural biology, in evolutionary biology, and in artificial intelligence, with more in the works. These topics are obviously very broad, and so are likely to be subdivided or otherwise revisited in future installments by authors with varying perspectives. Topics and authors will be chosen at the discretion of the editors along lines broadly corresponding to the usual content of this journal.

The author, having been asked to serve as Series Editor by the Editor-in-Chief, will endeavor to maintain a uniform flow of articles solicited from luminaries in the field. As a starting point to the series, I offer below a few vignettes and reflections on some longer-term influences that have shaped the discipline. I first consider the unique status of bioinformatics vis-à-vis science and technology, and then explore historical trends in biology and related fields that anticipated and prepared the way for bioinformatics. Examining the context of key moments when computers were first taken up by early adopters reveals how deep the roots of bioinformatics go.

## The Nature of Bioinformatics

Many who draw a distinction between bioinformatics and computational biology portray the former as a tool kit and the latter as science. All would allow that the science informs the tools and the tools enable the science; in any case, bioinformatics and computational biology are near enough cousins that their origins and early influences are likely to be commingled as well. Therefore, this article and series will construe bioinformatics broadly, bearing in mind it can thus be expected to have a dual nature. This duality echoes another that goes back to Aristotle, between “episteme” (knowledge, especially scientific) and “techne” (know-how, in the sense of craft or technology). The power of bioinformatics might be seen as arising from their harmonious combination, in the Greek tradition, lending it emergent capabilities beyond the simple intersection of computers and biology, or indeed of science and engineering.

### A Bioinformatics Revolution?

Many commentators refer to the “bioinformatics revolution.” If there has been one, was it a revolution in techne, like the Industrial Revolution, or in episteme, like the Scientific Revolution? Or was it both? The former suggests quantum leaps in scale and capability through automation, which seems to apply to bioinformatics almost by definition, while the latter implies an actual shift in worldview, raising a more philosophical question.

In Thomas Kuhn's famous conception of scientific revolutions, the early stages of paradigm formation are freewheeling and unstructured, while being effectively cut off from the pre-existing scientific milieu by their very novelty and an inherent incommensurability [1]. (The overused word “paradigm” can be excused in this context because it was Kuhn who instigated its overuse.) At some point, such “pre-science” becomes consolidated, establishes norms and templates, and settles into a “normal science” phase that allows for efficient discovery within a prevailing paradigm. Many would agree that the heady early days of bioinformatics had a makeshift feel, which has since matured into a more coherent, productive discipline with an established canon.

But before claiming the exalted status of a Kuhnian paradigm shift, it should be noted that Kuhn had in mind rather broader disciplines of science than bioinformatics, which was erected within and in relation to the comprehensive pre-existing scaffoldings of biology and computer science. To the extent that bioinformatics is a subsidiary or derivative field, it might call more for an evolutionary than a revolutionary model of development, of a sort some critics of Kuhn have advocated [2], [3]. From this perspective, its novelty and force perhaps derive from hybrid vigor rather than spontaneous generation, and it would seem to be more enabling than overturning—thus, primarily an advance in techne. Whether its rapid uptake and substantial impact qualify it as a technological revolution, or merely an evolutionary saltation, is perhaps only a matter of semantics.

In Kuhn's semantics, though, scientific revolutions produce profound shifts in our literal perception of reality. A computational perspective may radically change attitudes toward data, or even models of data, but it seems unlikely to fundamentally alter our sense of reality in the domain of biology. Still, true believers may argue that the “computational thinking” movement [4] as applied to biology, and perhaps even a view of life itself as a form of computation [5], does indeed rise to the level of a paradigm shift and a true revolution in episteme. We will explore a few such ideas below.

### The Role of Tools

A philosophical stance called realism essentially views episteme as independent of techne, holding that scientific truth is ultimately separable from how we measure or model it. But some assign tools a more prominent and persistent role. The Nobel laureate physicist P. W. Bridgman's influential notions of operationalism sought to reduce all scientific concepts to the literal means by which they are measured—that is, to operational definitions to be taken at face value rather than as describing some underlying idealization—so as not to over interpret or heedlessly conflate such concepts [6]. Thus, temperature would be defined in terms of thermometers rather than thermodynamics. Decades before computer scientists conceived of operational semantics and abstract data types, Bridgman considered a scientific concept “synonymous with the corresponding set of operations” [7]. Though controversial in physics circles, operationalization was seized upon by certain “soft” sciences like sociology as a way of achieving a more respectable exactitude.

The “hardening” of biology in the 20th century involved a reductionist convergence with chemistry and physics, enhanced by improving instrumentation, as well as new quantitative overlays to the legacies of Linneaus, Mendel, and Darwin. This often called for operationalizations, such as that of “enzyme” in terms of a measured activity, or that of the much-debated concept of “species” [3]. The practice predated but has lately been reinforced by bioinformatics. Computers, with their notorious literal-mindedness, require the same sort of “tightening up” of descriptive language as that urged by Bridgman [6], and have promoted ever more explicitly operational definitions, for example, of “gene”, in terms of the biological operations applied to DNA sequences [8].

Bridgman felt that by first recognizing clearly the distinction between operationally defined concepts such as gravitational and inertial mass, deeper insights like Einstein's equivalence principle would come more naturally. Today, operational definitions of biological concepts such as “gene” and “pathway”, distinguished as to whether they are probed by methods genetic, biochemical, or biophysical, are providing new insights as they are similarly integrated, with appropriate caution, by bioinformatic methods.

### The Instrumental Gene

Even scientific theories can be considered techne. Instrumentalism, an idea that goes back to the earliest days of the scientific revolution, takes a very pragmatic, almost mechanical view of theories, that they should be viewed merely as tools for predicting or explaining observations as opposed to directly describing objective reality [9]. Thus genetics was at first purely instrumental; regardless of any conviction that the gene had a physical basis, it was in practice a conceptual tool [10]. Instrumentalism doesn't ask whether a theory is true or false, but treats it as a sort of anonymous function taking data as input and producing predictions or explanations as output, the quality of which determine the appeal of the theory. Whether or not this is an adequate formulation of a scientific theory, it may be as good a definition as any of a bioinformatics application.

For a taste of the pre-molecular instrumental conception of genes, consider the moment in 1911 when Alfred Sturtevant made a key contribution. While still an undergraduate at Columbia University, he won a seat in the legendary “fly room” of T. H. Morgan's lab, which was busy identifying *Drosophila* mutants and counting offspring of various crosses. One day, upon realizing that multiple pairwise linkage strengths could not only be viewed inversely as distances but also collapsed onto a single dimension, he related that he “went home and spent most of the night (to the neglect of my undergraduate homework) in producing the first chromosome map” [11]. Long before the advent of bioinformatics, we nevertheless glimpse something of its “style” in this approach to data transformation, integration, and visualization—not to mention the fact that the youngest scientists often seem most adept at data-crunching (evidently even without benefit of a computer literacy surpassing that of their elders).

### Bioinformatics and Genes

The gene concept has undergone a steady evolution, in varying degrees instrumental and operational [12], [13]. The work of Barbara McClintock, for example, did much to ground the instrumental gene in physical locations on chromosomes by 1929 (though soon she in turn introduced instrumental notions of transposition and “controlling elements” that only became instantiated decades later in transposons, operons, and other regulatory apparatus, resulting in her belated Nobel Prize in 1983 [14]). Bioinformatics has played an increasingly important role in this evolution. Mark Gerstein notes that by the 1970s and 1980s, through a combination of cloning and sequencing techniques and then computational gene identification (whether by similarity or protein-coding signature), the working definition of a gene was reduced to a literal open reading frame of sequence—digitized data, in other words, critically dependent on electronic storage and algorithms—and that by the 1990s the gene had become for most practical purposes an annotated database entry [13]. Gerstein goes on to assert that the latest metaphor for genes is as “subroutines in the genomic operating system,” which suggests entirely new senses of operationalism and instrumentalism in biology, with a natural role for bioinformatics.

Yet operationalism and instrumentalism are often challenged in philosophical circles today, where they are considered to be “anti-realist” in their seeming disregard for the actual physical objects and processes underlying scientific concepts. In fact, it would appear that scientific progress is made when operational concepts are joined up, as by Einstein, or when instrumental concepts are mapped to successively more material forms, as by Sturtevant, McClintock, and eventually James Watson and Francis Crick. But this only bears out the functional utility of these “isms,” whose persistence suggests some underlying truth; they seem to wrestle with important concepts such as abstraction and reification (that is, concretization of abstractions as “first-class objects” for further manipulation) that are natural to and even promoted by the computational sciences. One thing they certainly assert is that it is a mistake to trivialize the role of tools in science as mere means to an end, as scientific ground truth may be hard to disentangle from those tools in the final analysis.

## Bioinformatics before Bioinformatics

Bioinformatics is far from being the first discipline to straddle the duality of episteme and techne. Mathematics is also considered a tool, vis-à-vis science, and here it is even more apparent how inseparable is the tool from the underlying scientific reality. Indeed, since Galileo and Newton, a common sentiment has been that science is never so successful as when its laws and explanations can be reduced to mathematical expression. Historically this had not been biology's forte, but early in the 20th century statistics and numerical analysis began to establish footholds in the field. Computers eventually carried these methods to new heights, though mainly by automating them rather than changing their underlying methodologies. Yet “pure” computer science is itself discrete math, separable from hardware, and soon this also would come to bear on a newly digital biology. As the following narrative suggests, the roots of bioinformatics may be detected in a mathematization of biology on many fronts, which machines only served to accelerate. The middle of the 20th century witnessed the key transitions.

### Mathematics Sets the Scene

The development of modern statistics was to a significant degree driven by its application to biology in the work of Francis Galton in the 19th century [15] and R. A. Fisher in the 20th [16]. Fisher helped put both Mendelism and Darwinism on a firm mathematical footing by 1930, and he is also credited with being the first to apply a computer to biology, albeit almost offhandedly. In a 1950 note giving tables of solutions to a differential equation developed for population genetics, Fisher says simply “I owe this tabulation to Dr. M.V. Wilkes and Mr. D.J. Wheeler, operating the EDSAC electronic computer.” [17] EDSAC, the Electronic Delay Storage Automatic Calculator, was built at the University of Cambridge Mathematical Laboratory; it is considered the first truly practical stored-program computer and the inspiration for the first text on computer programming in 1951 [18].

As biology became more quantitative throughout the 20th century, it increasingly assumed a “statistical frame of mind” [19]. In addition, naturalists adopted numerical methods for population modeling, and biochemists for enzyme kinetics; such applications remain the core topics of mathematical biology texts today. As noted, statistics and numerical analysis were considerably empowered by computers, but later these disciplines in turn contributed substantially to entirely new methods such as machine learning and multiscale mathematical modeling that are now central elements of bioinformatics.

Today's systems biology has a pedigree extending back at least to the first half of the 20th century. The biologist Ludwig von Bertalanffy began work on his holistic General System Theory then [20], while Norbert Wiener's cybernetics added an engineering math perspective in the 1950s encompassing feedback and regulatory systems that was influenced not only by early computer science, but also by evolutionary biology and cognitive science [21]. Network theory is often attributed to Gestalt social psychologists in the 1930s, but was productively merged with mathematical graph theory by 1956 [22].

Developmental biology began a long flirtation with math upon the publication in 1917 of D'Arcy Thompson's On Growth and Form, which was technically elegant and visually striking, albeit mostly descriptive [23]. Computing pioneer Alan Turing turned to biology during the tragic denouement of his life and was responsible in 1952 for a classic work in spatial modeling of morphogenesis [24], proposing a reaction-diffusion model of pattern formation that has only recently gained strong experimental support [25]. In this period Turing used the Manchester University Mark I, another trailblazing stored-program machine, to model biological growth in systems such as the Fibonacci patterns in fir cones described by D'Arcy Thompson [26]. Turing's labors on these problems are evident in page after page of calculations interspersed with dense machine code subroutines set down in his own hand, now archived at King's College, Cambridge [27].

### Turing's Legacy

Turing's bequest to biology is far more sweeping, though, insofar as bioinformatics would eventually embody a broad computational mathematization of the life sciences. The changes would be not only quantitative but also qualitative. As Fisher realized, “conventional” applications of numerical analysis could be taken to new levels, visualized as never before, and often freed from the necessity of closed-form solutions, by the sheer power of computers. But qualitatively, Turing's first efforts at biological computing began to shift the focus from the equations to the phenomena, from calculation to modeling. Moreover, Turing's overall legacy would soon foster a new perspective founded in discrete math, information theory, and symbolic reasoning, catalyzing trends that may already have been inchoate in the new molecular biology.

It is interesting to speculate whether Turing's turn toward biology, had he lived much past the discovery of the double helix, would have caused him to recognize and embrace this pivotal moment when biology became digital. He could not have failed to remark (as others soon would [28], [29]) how biological macromolecules incarnated his virtual automata, with biopolymers for tapes and enzymes to read and write them. Moreover, as a veteran of Bletchley Park and the wartime cryptanalysis effort, he might well have been drawn into the frenzy to decipher the genetic code that played out in the decade after his death.

In 1943 Turing had visited the US to share British codebreaking methods and met often with Claude Shannon, who was working on similar problems at Bell Labs [30]. Shannon's efforts on cryptanalysis were closely tied to his work in communication that, within the decade, would give rise to the new field of information theory. Turing took the opportunity to show him his 1936 paper on the Universal Turing Machine, since Shannon had been responsible in 1937 for the first rigorous application of Boolean logic as a formal basis for digital design, which to that point had comprised much more ad hoc arrangements of circuit elements. This contribution, which constituted Shannon's Master's thesis, is accorded great significance in the history of computing, but what has been all but forgotten is his 1940 PhD thesis, entitled “An Algebra for Theoretical Genetics” [31]. In this work, Shannon formalized population genetics just as he had circuit design, after spending an instructive summer at the Cold Spring Harbor Laboratory. Today it would be labeled bioinformatics.

One is left to wonder whether Turing and Shannon ever touched on biology during their lunchtime discussions. The geneticist James Crow feels that Shannon might well have extended his PhD work to have significant impact in the field but for the fact that he was drawn irresistibly to communication theory, first by the war and then by the lush technical milieu of Bell Labs [32]. It is intriguing to think that two giants of computer science and mathematics may have come so close to committing their careers to biology.

### Enter the Physicists

Instead it was physicists, some of them veterans of the Manhattan Project, who migrated to the new molecular biology and helped imbue it with their mathematical sensibilities. The attraction can be discerned in Erwin Shrödinger's famous wartime lectures and 1946 book *What is Life?* [33], which influenced Francis Crick and in turn was stimulated by the work of physicist-turned-biologist Max Delbrück, mentor to James Watson. In this slim volume, Shrödinger posits that chromosomes constitute Morse-like “code-scripts” of which “the all-penetrating mind, once conceived by Laplace, to which every causal connection lay immediately open, could tell from their structure whether the egg would develop … into a black cock or a speckled hen …” (pp. 20–21). Later, he suggests that some such executive in fact resides in the chromosomes themselves—that they are not only script but also machinery. This programmatic conceit, in itself strikingly evocative of Turing's self-referential automata and associated proofs, foretold the scramble to solve the puzzle of how the DNA sequence mapped to the other structures of life.

One of the first responses Watson and Crick had to their seminal 1953 paper was a letter from the physicist George Gamow, unknown to them, who 5 years before had proposed the Big Bang [34]. Gamow was already fascinated by biology, being friends with Delbrück and having published a popularization of a broad swath of science entitled *One Two Three…Infinity*, which included an exposition of fly genetics showing Morgan and Sturtevant's map [35]. Gamow's remarkable letter reimagined the DNA in each chromosome as a long number written in base four, so as to open up its analysis to number theory. He was soon calling this “the number of the beast,” suggesting that it varied only slightly among individuals, “whereas the numbers representing the members of two different species must show larger differences” [36]. Not only did Gamow thus neatly frame the future of sequence bioinformatics, but he went on to pose the question of the genetic code for the first time in purely formal terms—that is, in Crick's words, “not cluttered up with a lot of unnecessary chemical details” (quoted by Judson [30]). Postulating a collinearity of DNA with proteins (having seen Sanger's as yet fragmentary insulin sequences), the question for Gamow was how to “translate” the four-letter code to a 20-letter code.

Crick credited him with the simple combinatoric analysis that triplets of DNA bases would suffice [37], but Gamow seems almost to have recoiled from the prodigal degeneracy implied by the leftover information content (i.e., 4^3^ triplets for only 20 amino acids). Certainly Gamow's first model was overly complicated, involving as it did an overlapping and thereby non-degenerate code, as well as attempting to account for a direct translation from the DNA helix to the polypeptide by a physical docking [38]. (This perhaps reflects Shrödinger's errant instinct that chromosomes should be self-sufficient machines, or just enthusiasm for the astonishing implications of base pairing in the Watson-Crick model.) Still, Gamow set the game in motion, and served with great verve as its master of ceremonies.

### Codebreaking

A letter written in 1954 by Gamow to the biologist Martynas Ycas, preserved in the Library of Congress complete with marginal scrawls and cartoon drawings, suggests the tenor of the times: “After the collapse of triplet (major+2 minors) system a new suggestion was made by Edward Teller busy as he was with H bomb, and Oppenheimer. The idea is that each following aa. is defined by two bases … and the preceeding AA. Looks good! The ‘preceeding AA’ is characterized only by beeing [sic] ‘small’, ‘medium’ or ‘large.’ Last week I have discovered in Los Alamos the possibility of putting that system on Maniac, and this seems to be possible” [39].

What is most significant here is not the next ill-conceived model to which Gamow had turned, but rather the reference to MANIAC I, the Mathematical Analyzer, Numerical Integrator and Computer built to do weapons research by Nicholas Metropolis (of Monte Carlo fame) [40]. Once it was known that RNA directed protein synthesis, Gamow and Ycas did indeed use MANIAC to run a series of Monte Carlo simulations, first trying in 1954 to salvage overlapping codes, and when those proved untenable, testing in 1955 whether observed amino acid frequencies in proteins were likely to arise from non-overlapping triplet code translations [41]. (Metropolis also worked with others soon afterwards to computationally model cell multiplication and tumor cell populations [42], [43].)

These first MANIAC runs, requiring hundreds of hours, represent a new bioinformatics milestone, extending Turing's mathematical modeling of outward phenotypic patterns to stochastic modeling of the informational mechanics of life. As Lily Kay remarks, by “blurring the boundary between theory, experiment, and simulation … MANIAC had become the site of an artificial reality” [44]. Among the many scientists whom Gamow induced to take a run at the genetic code was Herbert Simon, who dabbled in this at the very moment he was beginning to apply computers to general problem-solving [44]. Simon would soon co-found the discipline of artificial intelligence, another fundament of bioinformatics, and another field deeply indebted to Turing. Gamow also recruited Robert Ledley, who in 1955 wrote a theoretical paper suggesting how computerized symbolic reasoning could apply not only to the genetic code but also to enzymatic pathways, portending modern pathway inference techniques [45]. Ledley went on to promote computer-based medical diagnosis and protein sequence tools and databases.

### The Urge to Model

The non-overlapping code Gamow and Ycas had arrived at by 1955 made an odd assumption, that the order of bases in each triplet was irrelevant. No doubt this was again motivated by a desire to dispose of degeneracy, as this scheme effectively did by collapsing permutation classes, but in some degree it may simply reflect the surrounding upheaval: biology was becoming an information science even as information science itself was aborning. After all, for the first half of the 20th century the prevailing mindset had been that DNA comprised repeating identical tetranucleotides, and that proteins were amorphous with no set linear sequence [46]. In his first letter to Watson and Crick, Gamow even suggested that genes were not localized, but smeared over the chromosome like a Fourier transform [34], his physicist's instincts flying in the face of all genetics since Morgan and Sturtevant. Gamow's biochemistry was initially just as naïve. He had scant basis to assume that exactly 20 amino acids were encoded, since others were known to occur naturally, if more rarely, and his first list of 20 actually included some of these and omitted valid ones [37]. Gamow's quantitative skills and fresh perspective were valuable and he learned quickly (much like computer scientists who came to biology later), but his concerted campaign to deduce the transcriptional and translational machinery on theoretical grounds seems a bit feverish in retrospect.

Even Crick was not immune, proposing a so-called “comma-free” code that utilized relatively few triplets as codons, but artfully chosen such that only one reading frame would be possible [47]. By chance, the math dictated that the capacity of such an unambiguous comma-free triplet code would be exactly 20 codons, making the theory immensely appealing—and dead wrong in the event. However, comma-free codes (as generalized to prefix codes) assumed great importance in computer science by way of Shannon's information theory, which strove to quantify, characterize, and ultimately ascribe utility to the very sort of degeneracy with which Gamow was contending [48]. While these theoretical excursions of Gamow and Crick foreshadow the future importance of Turing and Shannon to bioinformatics, they also exemplify how beautiful math, much less numerology, can run afoul of biological reality. Nowadays it is a truism that the bioinformatics should not get too far ahead of the data, yet we see that the instrumentalist urge to model is nothing new.

In fact, no amount of computational modeling or theory could by itself have discerned the full details of the genetic code, which by the early 1960s fell to bench scientists like the late Marshall Nirenberg to elucidate by means of cell-free translation systems and radioactive tracers. The US National Institutes of Health maintains in its archives pages from Nirenberg's lab notebooks, which include sprawling spreadsheet-like tables of hand-entered data, with multiple panels taped together and chaotically annotated [49]. It appears that he was literally drawing conclusions directly on the data sheets, outlining in red pencil the significant entries (as indeed might a cryptographer), such that the genetic code is seen emerging pictorially from the raw data. One senses that the carefully arrayed rows and columns of data, constituting an exhaustive all-against-all probe of triplet codes versus amino acids, was a harbinger of something new in biology; if it were done today, someone would no doubt label it the “codome.”

### Codifying Biology

Gamow's theoretical instincts were very much in the mold of Delbrück who, in his Nobel-winning 1943 paper with Salvador Luria, confirmed the basic tenets of Darwinism in bacteria through a profound interpretation of a trivial experiment [50]; to this end, they deployed reasoning that anticipated by 40 years the stochastic coalescent theory now prominent in population genetics and the analysis of polymorphism [51]. Physicists and statisticians brought to the biological table a degree of comfort with formalism, not only in math but also in language and logic, that would also typify computer science. A similar esteem for logic and formalism was also apparent earlier in the century in the philosophical movement called logical positivism, a major inspiration for Bridgman's operationalism [6], [9].

The logical positivists of the Vienna Circle between the wars felt that the time was ripe to reduce all of science (in fact all knowledge) to a pure empiricism, by which the only admissible statements would be those verifiable by direct observation. In the process they rejected all things metaphysical, and in fact felt that their efforts should go to serving science by following in its wake and providing a “rational reconstruction” of it in symbolic logic and formalized language. This entailed a strongly reductionist view of scientific theories and concepts, and faith in what Rudolf Carnap called the “Unity of Science” [52].

Today, when we codify biology in comprehensive formal ontologies, enforcing the stringent terminological and relational definitions demanded by computational structures, we are following in the footsteps of the Vienna Circle. We should take heed, because logical positivism did not survive the half-century. Among many critics, W. V. O. Quine attacked its reductionist tenets, holding that science is more like what he called a “Web of Belief” than a neat logic diagram, with complex interwoven structures creating mutually supporting bits of evidence and theory [53]. (One would be tempted to load it into Cytoscape.) Quine's views are more compatible with probabilistic networks and connectionism, and with the current assertions by systems biologists that the 50-year run of reductionism in molecular biology has played itself out [54]. Luckily, bioinformatics is adaptable.

### Computing Structures

Crystallographers were early adopters of computers in aid of their laborious calculations of Fourier syntheses and the like, beginning mainly with home-brew analog computers, but by the late 1940s gradually shifting to IBM punchcard tabulators programmed via plugboards (recognizable descendants of those used for the 1890 census) [55]. The first crystallographic applications of stored-program computers were done on EDSAC [56] and the Manchester Mark II [57] in 1952–1953. However, these were used for inorganic structures. The first application of computers to protein crystallography, which some consider the real forerunner of today's bioinformatics, was in fact for the first high-resolution structure, that of myoglobin, in 1958 [58].

By the 1960s, crystallographers were enthusiastic users of burgeoning computer technology, not just for the tedious core calculations but for many related routines as well; dozens of codes were written in the new FORTRAN and ALGOL programming languages, as opposed to being “hand-coded” at machine level [55]. This activity extended to visualization, including interactive molecular graphics first done by Cyrus Levinthal at the Massachusetts Institute of Technology, using an early time-sharing mainframe connected to an oscilloscope display of a wireframe model controlled by a prototypic trackball [59]. Of this, Levinthal wrote in 1966: “It is too early to evaluate the usefulness of the man-computer combination in solving real problems of molecular biology. It does seem likely, however, that only with this combination can the investigator use his ‘chemical insight’ in an effective way” [59].

Crystallographers went on to accumulate myriad structures and from these gained many “chemical insights” into life. Since the time of Sturtevant, geneticists as well had been doing mutant screens and maps that were undertaken not to test hypotheses in the first instance, but to gather grist for the mill of hypothesis generation. We tend to think of data-driven research as a recent innovation, and of the genome, proteome, and all the other “omes” as concepts uniquely enabled by technology, bioinformatics, and audacious scale. Indeed, omics is sometimes criticized as “high-tech stamp collecting” [60], but this could also have described Darwin's time on the *Beagle*. In fact, the groundwork for omics was laid long ago, and with it the data-rich, information-centric modality that came into its own with the advent of computers.

### Computing Traits

The first electronic computation of genetic linkage was performed by H. R. Simpson at the Rothamsted Experimental Station (where R. A. Fisher had created the statistical theory of experimental design) in 1958, on an early room-sized business model, the Elliott 401 [61]. However, as noted above and in a recent history by A. W. F. Edwards [62], this introduction of computers to genetics was merely the culmination of a continuous evolution from Mendel, through Morgan and Sturtevant, to Fisher and many other statisticians, theorists, and experimentalists.

The intellectual heirs of Linnaeus and Darwin were beginning to feel the influence of computing in this same period, spearheaded by math. George Gaylord Simpson, who perhaps most embodied the “modern synthesis” of paleontology, genetics, and evolution, showed by 1944 how the mathematics of population genetics pioneered by Fisher could relate to the fossil record [63], and brought a focus to evolutionary rates that presaged the molecular clock hypothesis central to modern phylogenetic reconstruction. Simpson had in 1939 co-authored the first book on quantitative methods in biology proper [64], and went on to devise operational metrics for ecologists to assess similarity of habitats based on the range of taxa found in them [65]. (Other statisticians provided estimators for species diversity within habitats [66], and ecologists were quick to adapt Shannon entropy to this purpose [67], as eventually would bioinformaticians for sequence motif analysis.) These were hand calculations as long as the data were limited to a few combinations, but when similarity metrics were adapted by others to classification of species based on increasing numbers of traits, the problem soon grew to become as onerous as had been the crystallographers' hand labors.

### Computing Trees

A phenetic operationalization of taxonomy (i.e., clustering by overall similarity) invited automation. In 1957, P. H. A. Sneath first applied a computer to classifying bacteria, using a relatively advanced Elliott 405 [68]; for readers not so equipped, he also showed how to simulate the computations by superimposing photographic negatives on which the data were encoded as transparent dots. The next year he published a follow-up with the wonderfully Tom Swiftian title “An Electro-Taxonomic Survey of Bacteria” [69]. Then, in 1960 an IBM staff mathematician, Taffee Tanimoto, worked with David Rogers of the New York Botanical Garden to apply computers to plant classification [70]. (Their similarity metric, bearing Tanimoto's name, is commonly used today in cheminformatics to compare compounds; in fact, by 1957 there had already been amazingly advanced work done on computational chemical structure search by the National Bureau of Standards for the US Patent Office [71].) Though the idea of quantifying relationships went back to the previous century, computers thus helped to precipitate the new field of “numerical taxonomy” with the appearance of the 1963 book of that name by Sneath and Robert Sokal [72], which also broached the idea of extending numerical approaches to phylogeny.

As related by Joel Hagen [73], computational research in classification soon came to be driven by biological systematics with its very large datasets of well-studied characteristics, an existing classification system for reference, and cladistic methods with explicit rules and formal logic for establishing evolutionary histories (despite the tension between pheneticists and cladists, which is still evident in bioinformatics today). In return, computers had a prodigious effect on systematics, shaping the mathematics used, promoting formality of methods, and most importantly, enabling the molecular systematics that was about to explode on the scene. In a few short years, with the work of Dayhoff, Fitch, and many others, protein structures and evolutionary trees would come together in a powerful synergy that still informs much of bioinformatics.

Sneath later recollected that population biologists proved open to numerical taxonomy (though Fisher, characteristically, worried that it didn't have an exact statistical basis), while evolutionary biologists were at first more dubious [74]. Traditional taxonomists felt most threatened of all; David Hull tells of a contentious meeting where one indignantly asked, “You mean to tell me that taxonomists can be replaced by computers?” and was answered, “No, some of you can be replaced by an abacus” [3]. G. G. Simpson himself was receptive but, realizing the tectonic shift that was at hand, was almost wistful in addressing his colleagues (quoted in [73]): “We may as well realize that the day is upon us when for many of our problems, taxonomic and otherwise, freehand observation and rattling off elementary statistics on desk calculators will no longer suffice. The zoologist of the future … often is going to have to work with a mathematical statistician, a programmer, and a large computer. Some of you may welcome this prospect, but others may find it dreadful”.

## The Bioinformatic Synthesis

Despite Simpson's ambivalence, the most salient feature of the development of bioinformatics has been its success as an interdisciplinary enterprise. The combination of biology and computer science seems increasingly to be syncretic rather than eclectic—not simply one of juxtaposition and coexistence, but a substantial merging of systems with different worldviews, methods, and cultures. At an even more fundamental level, beyond any disciplinary boundaries, it represents a successful synthesis of episteme and techne.

At first, it may have appeared more like a marriage of convenience than of true minds. Notwithstanding the examples cited above, much of the early adoption of computation by biologists was for purposes of laboratory information management, with little sense that it would ever be good for more than straightforward data acquisition, reduction, and storage. By the same token, theoretical computer scientists who first encountered biology sometimes seemed less interested in nature than in citing motivating examples for string algorithms or combinatoric problems with little regard for their practical application. Happily, as with the mutual stimulation between biological taxonomy and computational classification methods, the subsequent history of bioinformatics took a decidedly more syncretic turn, often as a result of felicitous collaborations.

Even when individuals are willing, institutions and policies can make or break cross-disciplinary studies, in any field. Carnap, the logical positivist, undertook advanced training in both physics and philosophy, and wrote a doctoral thesis at the University of Jena on an axiomatization of space-time. Both the physics and philosophy departments found the work interesting, but as a dissertation both turned it away, each saying it was more pertinent to the other field. A no doubt exasperated Carnap rewrote it with an undeniable philosophical cast and received his degree from that department without further ado in 1921 [75]. Many who entered bioinformatics only a few decades ago might empathize, but it hardly seems an orphan discipline today, with major funding initiatives, training programs, and entire institutes devoted to it.

For reasons such as these, a retrospective view of the roots of bioinformatics is likely to be a social history as much as anything, tracing the interaction of scientific disciplines down to the level of university environments, scientific conclaves, individual collaborations, and networks of interaction. Indeed, the importance of the sociology of science to its progress is considered one of the main intellectual legacies of Kuhn's work, even discounting his theories of scientific revolution [3], [9].

The tentativeness and doubt voiced by pioneers like Levinthal and Simpson have faded. The insights of Fisher, Turing, and Shannon now underpin the standard repertoire of bioinformatics tools. The theoretical intuitions of Delbrück and Gamow drive those tools, and the empirical sensibilities of Sturtevant, McClintock, and Nirenberg are embedded in them. Whether this is revolution or evolution, the story of how it came to pass—the roots of bioinformatics—should make compelling reading.
